# Incorporating positive youth development into the therapeutic model for incarcerated young woman

**DOI:** 10.15761/fwh.1000135

**Published:** 2018-01-10

**Authors:** Diane L Elliot, Leslie D Leve, Kristi H Racer

**Affiliations:** 1 Division of Health Promotion & Sports Medicine, Oregon Health & Science University, USA; 2 Prevention Science Institute, University of Oregon, Eugene, USA; 3 Senior Research Analyst, Oregon Youth Authority, USA

**Keywords:** adolescents, emerging adults, juvenile justice, Positive Youth Development, sleep, physical activity

## Abstract

Young women in the juvenile justice system have high rates of prior physical and sexual abuse, substance use and psychiatric disorders. Understandably services usually are based on a therapeutic model to address those needs. Positive Youth Development (PYD) is a complementary alternative format that aims to provide resilience, life competencies, and self efficacy for pro-social actions. We provide a narrative review of PYD programs with a focus on how those relate to youth in closed custody. Sleep and physical activity are two behaviors where incarcerated young women still have personal agency, and we present the additional relevance of those program aspects. We describe methods and findings from a feasibility trial of an existing evidence-based, peer-led program for young women high school athletes used with incarcerated young women. Findings are placed in the context of established models of behavior change. The program was feasible and acceptable, and in this small trial, results demonstrate the format’s potential efficacy. PYD may provide a trajectory of success and assets that could durably assist these young women following incarceration.

## Introduction

Young women in the juvenile justice system are a high-risk, vulnerable population largely hidden from public view [1]. Yet, they comprise almost 30 percent of youth in the juvenile justice system [2]. The typical female offender is low risk but high need. That is, unlike their male counterparts, most are remanded to custody for less serious crimes [1], and they have significantly greater rates of physical, sexual, and emotional abuse, with greater odds of depression, post-traumatic stress disorder, and anxiety [3]. Most have a history of substance use, with rates five-times the general population [4]. The future for these young women often includes ongoing victimization plus alcohol and other drug use [5]. As a group, young women in the juvenile justice system have been characterized as the “most medically underserved of all adolescent populations” [6].

Existing services for young women in the juvenile justice system are not optimal. The 2015 report, Gender Injustice: System-Level Juvenile Justice Reforms for Girls, noted, “juvenile justice systems are routinely failing to modify promising system reforms for girls or even to collect data on how girls are affected by the problems systems seek to remedy” [7]. Evidence-based therapeutic programs are available for incarcerated youth; such as Aggression Replacement Training, Dialectical Behavioral Therapy and Multimodal Substance Abuse Prevention [8–10]. However, those programs generally are for delivery in family or community settings, rather than during incarceration, and they are aimed at specific risky behaviors or disorders [11]. Nationwide less than 10 percent of eligible high-risk juvenile offenders receive evidence-based treatment [12–14].

Around 2000, a new paradigm of “Positive Youth Development” (PYD) entered the literature [15], where the focus shifted from managing adolescents’ problems to developing their assets [16–18]. The existing therapeutic model is understandable given the extensive needs of incarcerated young women. However, augmenting a therapeutic model with PYD could enhance, not replace, current services. We present a narrative review of PYD and a rationale for its use related to physical activity and sleep, two domains where incarcerated young women still have personal agency. We report findings from a pilot PYD program with incarcerated young women and suggest next steps for adding PYD to the care of these offenders in closed custody.

## Positive youth development

The Interagency Working Group on Youth Programs is a collaboration of 20 youth-supporting U.S. federal agencies, which defined PYD as an intentional, prosocial approach that respectfully engages youth to recognize, utilize, and enhance their strengths and that promotes positive outcomes [19], such as resilience, prosocial norms, behavioral competencies, positive self-identity and enhanced self-efficacy [20,21]. Rather than a specific curriculum, PYD applies a set of practices to a program, such as a respectful, engaging and supportive learning environment; opportunities for communication among participants and with teachers/leaders; and a structure to build personal competencies [22,23]. Often the assets developed by PYD programs are termed the five C’s: competence, confidence, connection, character and caring. Those have become so engrained that instruments have been developed to assess those specific dimensions [24]. With these assets, environmental risk factors are less influential and/or have fewer adverse consequences. Unlike a therapeutic approach, which aims to remedy a problem or focuses on risky behaviors, PYD develops various positive assets and is described as a “strength-based” approach [25].

PYD programs assist adolescents’ reflection on existing behaviors, selecting personal goals, and pursuing healthy and/or prosocial actions by applying available resources [26]. PYD’s goal setting process enhances intentional self-regulation, which builds skills to manage one’s actions [27–29]. The last component parallels aspects of self-determination theory’s personal growth, where positive development includes self-efficacy for actions, empowerment to make choices, and a feeling of social connection [30].

PYD originally was applied to universal programs for youth in community settings. Perhaps the best known and well-studied PYD format is the 4-H program. A longitudinal evaluation of local 4-H programs involving approximately 4,000 youth from nearly all states, concluded that youth consistently engaged in 4-H were at much lower risk of having personal, social, and behavioral problems than other youth [31]. Subsequently, PYD programs have been developed for specific segments of the young adult population. For example, PYD programs have been beneficial for youth with chronic medical problems [32].

PYD programs also can target specific behaviors and actions. Reviews of PYD programs suggest that these efforts can positively impact actions relevant to justice involved youth, including reducing violence, harmful sexual practices and drug use [33–35]. A systematic literature review identified 15 PYD programs with evidence of promoting adolescent sexual and reproductive health, including the prevention of teen pregnancy and sexually transmitted infections. The level and duration of the impact health outcomes were substantial, with the impact of several programs extending into adulthood [36]. Having strengths and assets has been shown to be inversely related to problem behaviors including substance use, aggressive behavior, and violence [27,37]. Accordingly, enhancing key assets in youths may be a viable approach to reducing delinquency while fostering healthy development.

Young women in the juvenile justice system might be especially appropriate candidates for a PYD program. Because of their high prevalence of abuse and risky sexual experiences, their childhoods end early, and the transition to autonomy is accelerated [38]. Accordingly, PYD programs could offer this population an unmet opportunity for building decision making skills and help them develop personal agency to use their autonomy in pro-social ways [39].

## Personal agency while incarcerated: Sleep and physical activity

If a critical component of PYD programs is practicing healthy self-regulation, how is that possible in the tightly controlled setting of incarcerated youth? Both adequate restorative sleep and regular physical activity are behaviors amenable to goal setting, behavior change and positive reinforcement for achieving those goals while incarcerated. Each is an action that also may be especially beneficial for young women in the juvenile justice system.

Chronic sleep deprivation is common among teenagers, especially young women [40]. In a large U.S. survey, sleep disorders were twice as common among adolescent girls compared with boys, with almost one-third of females experiencing problems initiating or maintaining sleep [40]. Getting less sleep did not reflect needing less sleep, as the consequences of inadequate sleep also were increased. A recent report indicated that 20 percent of young women complain of excessive daytime fatigue, a rate three-times greater than male counterparts [41]. Lack of sleep is not just related to teens’ staying up late and awakening early for school; insomnia also is a problem among adolescents. In four large prospective studies, insomnia has been causally linked to increased risk of unhealthy behavior, including smoking, alcohol and other drug use, drunk driving, and carrying a gun [42–45].

Growing evidence suggests that adequate sleep plays a crucial role in an adolescent’s healthy development, particularly in the regulation of behavior, emotion and attention. The ability to do different tasks simultaneously, such as combining cognitive and emotional tasks, may be particularly vulnerable to the effects of sleep loss. Dahl and Lewin have suggested that multi-tasking is one of the developmental milestones that adolescents encounter, and without adequate sleep that ability is impaired [46]. Prospective studies have demonstrated that sleep problems and/or sleep deprivation increase the risk for subsequent mental/emotional dysfunction and high rates of risk taking behaviors in adolescents [42,47–51].

Sleep has been largely overlooked among researchers interested in adolescent delinquency. In a cross-sectional study using data from the National Longitudinal Study of Adolescent Health, youth who typically slept seven or fewer hours per night reported significantly more property delinquency than those achieving the recommended 8 to 10 hours per night. And those getting five hours or less reported significantly more violent behaviors. Overall getting inadequate sleep was associated with three times the level of adverse outcomes [52].

Detention facilities often have a lights-out time established by staff. However, that does not mean that hours of sleep are established for these young women. They may have the option to read in their beds, and those with insomnia may not go to sleep at lights out. In other settings, improving sleep habits has helped in managing both depression [53,54] and PTSD [55]. Easily implemented sleep improvement programs, adaptable for young women in correctional facilities, also are also beginning to be used with young adults [56]. Those features could be incorporated into a PYD program for incarcerated young women.

Physical activity is a second domain in which youth in closed custody can have some control. Participating in regular physical activity yields a wide range of well documented physical and psychological [e.g., increased self-esteem, self-competence, improved body image, reduced depressive symptoms, and reduced issues with post-traumatic stress] benefits [57–62]. Regular exercise may be more important for young women than men, as females’ physical activity levels decline more than males’ during adolescence [63]. In addition, strength training may have heightened benefits for this group, as it has been reported to increase women’s self-esteem [64]. Furthermore, it has been suggested that participation in physical activity is linked to enhanced brain structure and function, cognition, and academic performance, via direct and indirect physiological, cognitive, emotional, and learning mechanisms [65]. Exercise’s psychological benefits are especially relevant for adjudicated young women (e.g., increased self-esteem, improved body image, reduced depressive symptoms and stress reduction) [57–62]. In addition to direct personal benefits, PYD physical activity programs have demonstrated that group exercise sessions when structured to facilitate positive assets offer the opportunity to build social skills by providing a context that requires conflict resolution, cooperation, and, goal setting [66–69].

In other contexts, physical activity has been a component of PYD programs. Positive Futures is a government-funded PYD physical activity-based intervention in England, where physical education is not part of the routine school curriculum. When assessed, that program favorably impacted at-risk youth by assisting in developing self-esteem, a sense of control, and decision-making skills [70]. The PYD link to regular exercise and self-control abilities also has been assessed specifically among young women. Self-control abilities are thought to depend on a person’s resources, and when a situation demands two consecutive acts of self-control, performance on the second one can be impaired, either due to lack control or its depletion. The second factor may contribute to why crime, violent acts and addictive relapses tend to occur later in the day, a phenomenon possibly attributed to a depletion of self-control resources after completion of daytime activities [71]. When studied among college age women, aerobic exercise appeared to enhance self-control abilities [72].

## PYD in a locked setting: A feasibility trial

The starting point for a trial of a PYD informed program among incarcerated young women was an existing evidence-based program for female high school athletes called, ATHENA (Athletes Targeting Healthy Exercise & Nutrition Alternatives). ATHENA is a gender specific, peer-led team-centered harm reduction/health promotion curriculum, which is integrated into a school sport team’s usual practice activities. In a randomized trial, it enhanced positive assets (exercise self-efficacy, controlling mood, refusal skills, healthy norms, and resisting media pressure) and deterred drug use and other health-harming behaviors [73–75]. Subsequent long-term follow-up of ATHENA program participants indicated durable positive changes. One to three years following high school graduation, compared with control athletes, intervention-ATHENA graduates reported significantly less lifetime use of cigarettes, marijuana and alcohol, with improved nutrition behaviors and healthier self-image [75]. While from the athletes’ perspective, ATHENA is about better individual and team performance, its broader curriculum related to communication abilities, decision making, self-esteem, avoiding negative emotions and resisting unhealthy societal pressures.

For high school students in general, health or physical education is neither part of the educational “Common Core” nor required for high school graduation [76,77], and as such, it generally is not an education offering provided to incarcerated youth. The Oregon Youth Authority (OYA) is an independent state agency that serves Oregon’s most delinquent youth ages 12 through 24. The majority of these young adults are on probation or parole, while approximately 40 percent live in one of OYA’s closed-custody facilities, with an average length of stay of 8 months. Incarcerated youth who have not graduated from high school attend classes toward getting their degree. However, that content does not include “health” or “physical education.” The staff of the OYA strives to incorporate PYD approaches in its youth services, and they conducted a pilot implementation of ATHENA plus adding a weekly workout session at its facility for young women.

Despite being designed for high school athletes, the ATHENA curriculum had features that facilitated its use at a correctional facility. Designed to be used by busy coaches during the sport season, it is explicitly scripted, which makes delivery easy for both counselors (filling the coach’s role) and peer-leaders. Both only required orientation to the leader manual and workbook format. Prior research demonstrated that the use of peer-leaders adds to the PYD component, as they provided more relevant examples, improved their communication skills, enhanced self-efficacy and better reinforced content through ongoing contact [78,79]. In addition, with peer-leaders, because actions are prompted, modeled and valued by others in the group, the social persuasion for change may be better internalized, enhancing durability of new abilities [80].

## Participants and methods

The OYA conducted a pilot implementation of OYA ATHENA in one facility with 12 volunteer participants [mean age 17.2 years, range 16 to 18]. The sessions used the original ATHENA program for high school athletes with minimal modifications, as this was staff’s preference in order to see how the format worked, while acknowledging the content’s limitations. As originally designed, ATHENA sessions run approximately 45-minutes, and one session is done each week of the sport season for 8 total sessions. Thus, the content could be easily worked into OYA usual activities, and it was completed at the facility in 10 weeks.

The trial was designed to assess feasibility and acceptability. Sessions were observed, and participants and staff debriefed after the fourth and final sessions. In addition, before and following the program, participants completed a brief survey containing 27 items related to their knowledge, attitudes and behavioral intentions. The final survey also contained global ratings concerning the program. Individual items used a 7-point Likert agreement scale, and items were combined into risky and healthy attitudes/intentions constructs (Table 1).

## Results

**Figure 1.**
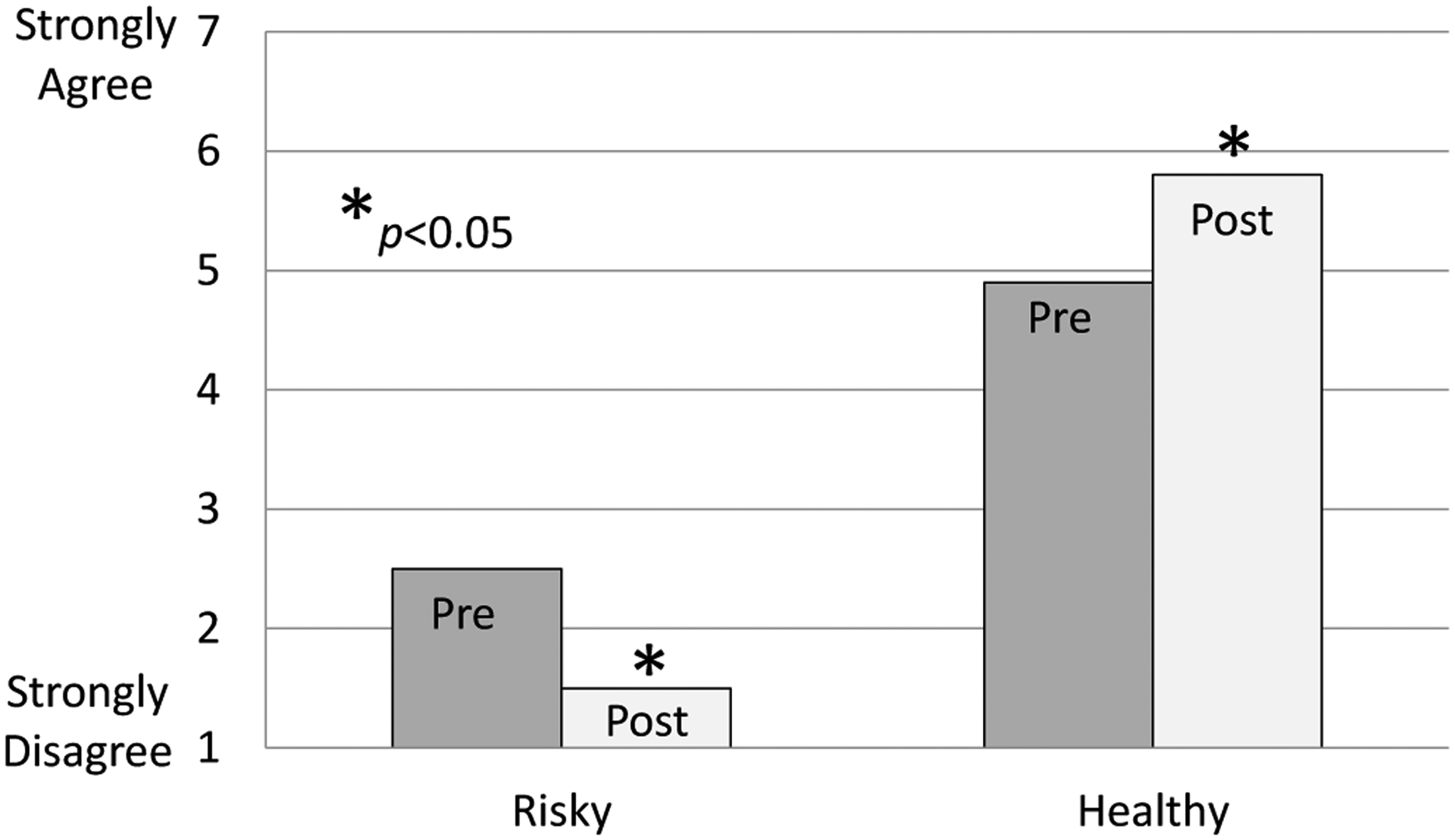
Changes in the Risky and Healthy constructs following the OYA-ATHENA program.

Eight participants completed the program, with attrition due to release from the facility for four participants. Survey responses demonstrated a positive program impact. The risky behavior construct was significantly reduced, and a healthy behavior construct increased (Figure 1). Global ratings also were uniformly positive, with participants’ mean rating greater than 6 for recommending the OYA-ATHENA to others. The program was feasible and acceptable to participants, and with additional minor modifications, the staff have continued to use the program.

## Conclusions and next steps

The peer-led scripted format was acceptable to participants and feasible for delivery. Its focus on active learning and personal goal setting in the context of mutual support embody the structure of PYD programs. However, the ATHENA curriculum scope and sequence were developed from a cross-sectional study of the needs of young women high school athletes [74]. Incarcerated young women require a curriculum designed for their specific needs. Building that program also necessitates an understanding of what aspects of PYD principles are most important in constructing the program. While the potential benefits of PYD programs are well established, the critical components causally related to its positive outcomes have not been defined. Others have noted the importance of assessing the specific processes of effective PYD programs to move the field forward [80]. Mediation analysis is a means to assess the hypothetical relationships among intervention components, targeted variables, and outcomes. It provides an explicit check on whether a program affected the intermediate variables and whether the purported theoretical relationship of those variables to outcomes holds [83–85]. In our prior randomized trials of school [including the ATHENA program] and worksite-based health promotion/harm reduction interventions, we have applied mediation analysis to establish a model of behavior change [85]. To date mediation analysis has not been applied to PYD programs, and the mechanisms of action are based on existing theories and identification of shared features of effective interventions [86]

In 2016, Bonell and colleagues reviewed 16 PYD programs relating to substance abuse and violence, with the goal of identifying the theoretical underpinnings of programs’ positive outcomes [86]. The synthesis suggested that PYD provided skill training to enhance intentional self-regulation, and that practicing that skill generalized to the betterment of participants. In addition, a second PYD program mechanism to emerge was the concept of buffering, by which youth develop assets that protect them from environmental risks. For example, having a positive self-identity might reduce susceptibility to negative peer influences [87].

**Figure 2.**
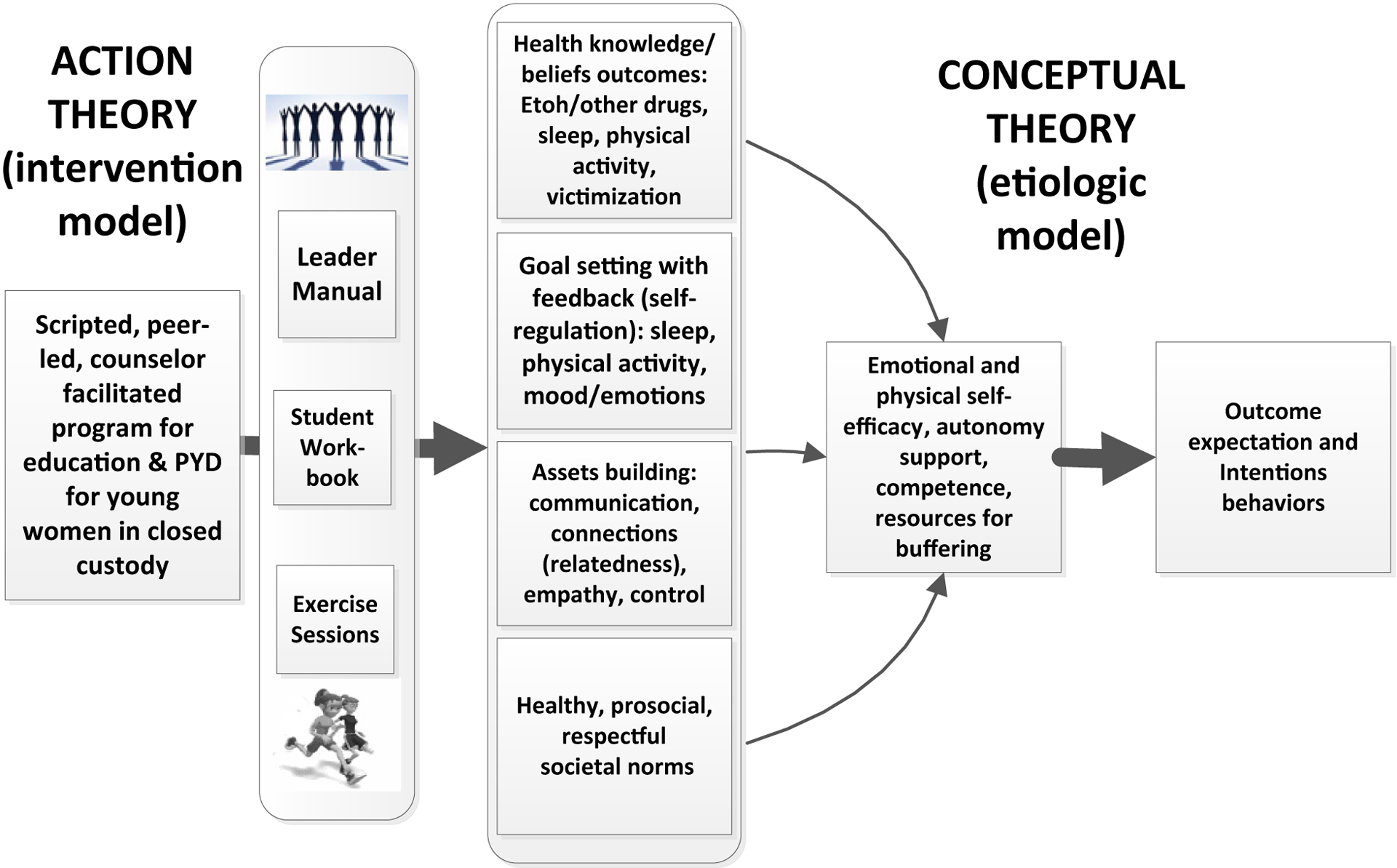
Proposed PYD program model showing the intervention impacting purported mediators (Action Theory), and those mediators influencing outcomes (Conceptual Theory).

A hypothesized model of a PYD program for incarcerated young women is shown in Figure 2. It begins with our established model of similar peer-led, group-centered curricula, including an analysis of the original ATHENA intervention [85, 88]. And, it includes superimposed features from processes of PYD programs and the focused self-efficacy behaviors related to achieving appropriate sleep and attending a program of regular exercise tailored to the setting (Figure 2). Because such a program, once developed, would be easily scalable and include content not usually addressed in current therapeutic programs or educational offering, it potentially could achieve reach into many closed facilities for young women.

In summary, although limited in its size and scope, the current feasibility study of the OYA-ATHENA program provides preliminary evidence of the feasibility of and likely value in implementing PYD programs focused on health behaviors within a correctional facility for young women. Others have advocated for applying PYD to the juvenile justice system, suggesting recasting it as “Positive Youth Justice” [89]. Those authors also noted the absence of existing programs and the “uncharted pathway from theory to practice” [89]. Although limited in size and scope, the current trial provides feasibility, acceptability and likely value of a PYD program focused on healthy behaviors for young women in closed custody. It provides an initial sign post for the development and assessment of PYD programs in this setting.

## Figures and Tables

**Table 1.** Risky and Healthy Constructs.

**Risky Survey Items**
If I were to use diet pills, they probably wouldn’t have any bad effects
In the future, I would consider using diet pills or energy pills to lose weight
In the future, I would consider using drugs to control my weight
In the future, I would consider vomiting to lose weight
The bad effects of bodybuilding steroids go away as soon as you stop using them
Only a few people who use bodybuilding steroids ever have harmful or unpleasant side effects
If I were to use bodybuilding steroids, they probably wouldn’t have any bad effects
Most products advertised in magazines do what they say they do
Ads in magazines are based on science and are usually true
**Healthy Survey Items**
I am aware of the calories, fat, and protein in the food I eat
I keep track of the calories, fat and protein I eat
What I eat is important for my athletic performance
I know the basics of good nutrition
I know how to train with weights to increase my strength
I would be comfortable turning down a friend who offered me bodybuilding steroids
I would be comfortable turning down a friend who offered me alcohol or drugs
I know how to turn down someone wanting me to skip meals or use drugs to other unhealthy behaviors to lose weight
I know how to control my mood or emotions
In general when you do more fun things, your mood is better
